# Temporal hemiarthroplasty for distal femoral osteosarcoma in early childhood: a case report

**DOI:** 10.1186/s12957-020-02047-8

**Published:** 2020-10-28

**Authors:** Jungo Imanishi, Masayuki Tanabe, Taisei Kurihara, Tomoaki Torigoe, Jun Kikkawa, Atsuhiko Ohta, Atsuko Watanabe, Ryuhei Tanaka, Kazuo Saita, Yuho Kadono, Yasuo Yazawa

**Affiliations:** 1 Department of Orthopaedic Oncology and Surgery, Saitama Medical University International Medical Center, 1397-1 Yamane, Hidaka, Saitama, 3501298 Japan; 2 Department of Orthopaedic Surgery, Saitama Medical University Hospital, 38 Moro-hongo, Moroyama, Saitama, 3500495 Japan; 3 Department of Orthopaedic Surgery, School of Medicine, Teikyo University, Tokyo, 1738605 Japan; 4 Department of Orthopaedic Surgery, Saitama Medical University Saitama Medical Center, 1981 Kamoda, Kawagoe, Saitama, 3508550 Japan; 5 Department of Critical Care and Emergency, Saitama Medical University International Medical Center, 1397-1 Yamane, Hidaka, Saitama, 3501298 Japan; 6 Department of Pediatric Hematology and Oncology, Saitama Medical University International Medical Center, 1397-1 Yamane, Hidaka, Saitama, 3501298 Japan

**Keywords:** Osteosarcoma, Younger children, Limb-sparing surgery, Temporal hemiarthroplasty, Partial cementation, Periosteum, Masquelet technique, Induced membrane

## Abstract

**Background:**

Prosthetic reconstruction for distal femoral osteosarcoma is challenging for younger children. We herein report a successful case of limb-sparing surgery for a younger patient with distal femoral osteosarcoma requiring osteo-articular resection.

**Case presentation:**

A 5-year-old girl with high-grade conventional osteosarcoma in the left distal femur underwent a series of surgeries. After three cycles of neoadjuvant chemotherapy, limb-salvage surgery was planned because femoral rotationplasty had been refused. At 6 years and 2 months old, distal femoral resection and temporary spacer insertion using a 7-mm-diameter intramedullary nail and molded polymethylmethacrylate was performed. At 7 years and 8 months old, secondary surgery was performed because the first spacer had been dislocated and the residual femur became atrophic. The distal end of the residual femur was removed by 1 cm, but the periosteum and induced membrane around polymethylmethacrylate was preserved. In order to stabilize the spacer against the tibia, a custom-made ceramic spacer with a smooth straight 8-mm-diameter stem was utilized. The bone-spacer junction was fixed with polymethylmethacrylate and then covered with the preserved periosteum and induced membrane. After surgery, the bone atrophy improved. At 9 years and 7 months old, the second spacer was removed because it had loosened, and the knee joint was reconstructed using a custom-made growing femoral prosthesis with a curved porous 8.5-mm-diameter stem. Cancellous bone tips from the proximal tibia were grafted around the bone-prosthesis junction underneath the induced membrane. At 10 years and 5 months old, the patient was able to walk unsupported and a radiograph showed further thickening of the cortex of the residual femur without any stress shielding. Although having 5 cm of limb length discrepancy, the patient and her mother were satisfied with the function. The MSTS score was 24 out of 30 points. Repeated limb length extensions are planned.

**Conclusions:**

This case report provides an example of limb-salvage surgery after distal femoral resection in a small child. The use of a temporary spacer utilizing partial cementation and preservation of the periosteum and induced membrane appears to afford a viable limb-salvage option after distal femoral resection for younger children.

**Supplementary Information:**

**Supplementary information** accompanies this paper at 10.1186/s12957-020-02047-8.

## Background

The distal femur is the most common location for osteosarcoma in childhood. Although limb-sparing surgery has become the norm in the treatment of osteosarcomas in the extremities, it is still challenging for younger children. Reconstruction options after osteo-articular resection of distal femoral osteosarcoma in children include femoral rotationplasty, osteo-articular allograft with or without prosthetic reconstruction, distraction osteogenesis, and mega-prosthetic reconstruction [1–6]. Decision-making with regard to surgical intervention is based on the consideration of the patient’s lifestyle, the preference of the patient/guardian, and the accessibility of medical resources.

In most previous reports, prosthetic reconstruction for the distal femur was applied for children aged 6 years or older, presumably due to the fact that femoral intramedullary diameter in younger children is too narrow to allow insertion of a durable stem. We herein report the case of a 5-year-old girl with distal femoral osteosarcoma treated with temporal hemiarthroplasty followed by expandable mega-prosthesis reconstruction, focusing on our unique surgical tips and ideas for temporary hemiarthroplasty.

## Case presentation

**Fig. 1:**
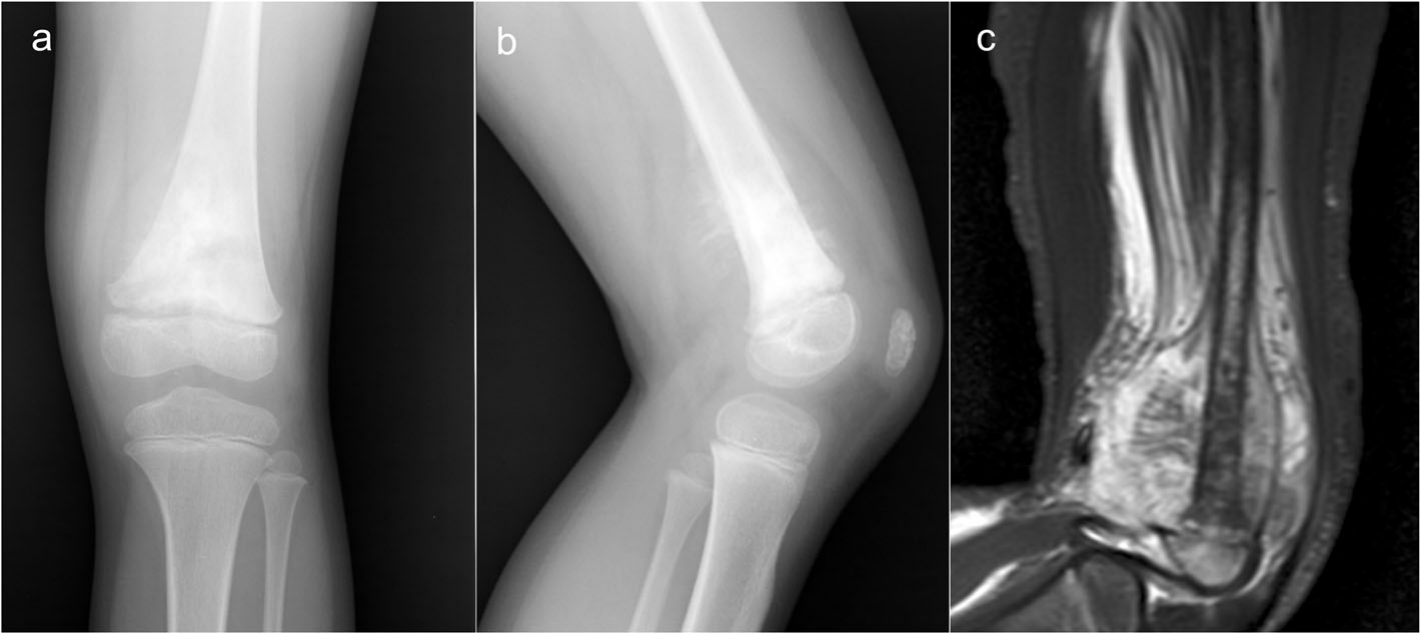
Radiographic findings at first presentation: **a**, **b** radiographs of the left knee showing an irregular abnormal periosteal reaction, and **c** a STIR MR image showing massive extra-osseous tumor extension and involvement of the epiphyseal plate in the distal femur

**Fig. 2:**
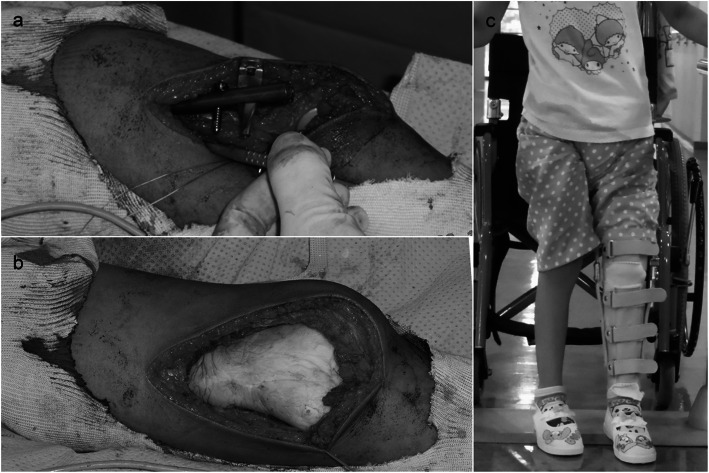
**a**, **b** The temporal spacer made of a humeral nail and molded PMMA, **c** weight bearing on the affected leg using a brace, and **d**, **e** post-operative radiographs

**Fig. 3:**
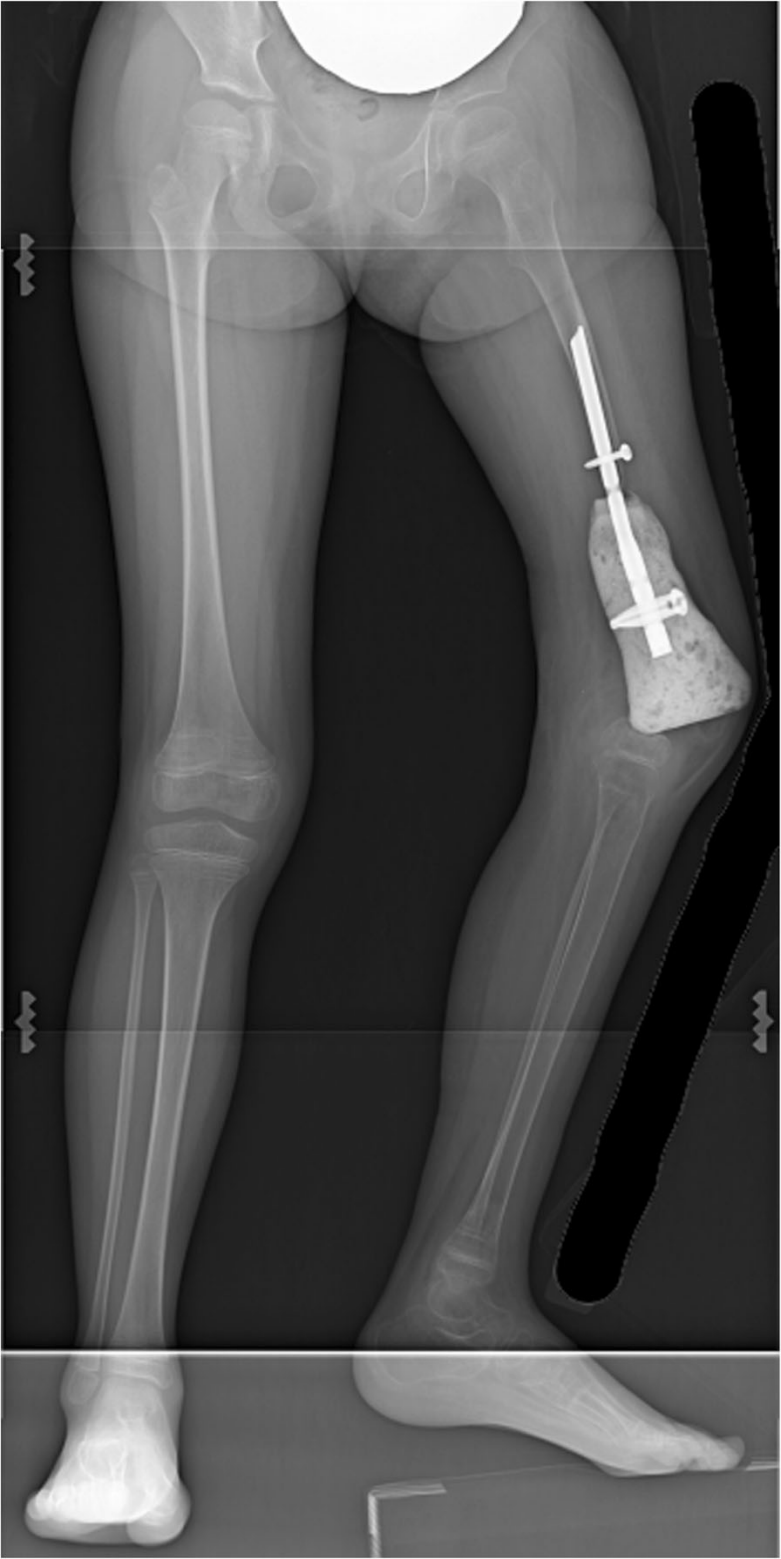
A radiograph showing anterior dislocation of the spacer and bone atrophy

A 5-year-old girl initially presented to our department due to worsening pain in the left proximal thigh. Osteosarcoma was suspected radiographically (Fig. 1). Open biopsy revealed a high-grade conventional osteosarcoma. The tumor was initially staged as IIB (T2b N0 M0), and neoadjuvant chemotherapy was commenced in accordance with the NECO-95J protocol [7]. After three cycles of neoadjuvant chemotherapy, the pain was alleviated, the extra-osseous mass was dramatically reduced, and limb-salvage surgery was planned. As the tumor had already invaded the epiphyseal plate and epiphysis of the left distal femur at initial presentation, osteo-articular resection was thought necessary so as to obtain a negative surgical margin. At 6 years and 2 months old, distal femoral resection and temporary spacer insertion was performed (Fig. 2a, b). Both medial and lateral meniscuses were preserved. The spacer was hand-made, using an intramedullary nail of 7 mm in diameter and molded polymethylmethacrylate (PMMA). Because there were no commercially available molds fitting the distal femur of such a young child, we made the spacer by hand, imitating the original distal femur of the patient that had been just removed. After surgery, the left leg was externally fixed in a cast for 4 weeks. Partial weight bearing on the affected leg, supported by a hinged knee brace, was thereafter commenced (Fig. 2c); however, the spacer was dislocated at 7 months post-operatively, preventing the patient from bearing weight and leading to bone atrophy in the left leg (Fig. 3).

**Fig. 4:**
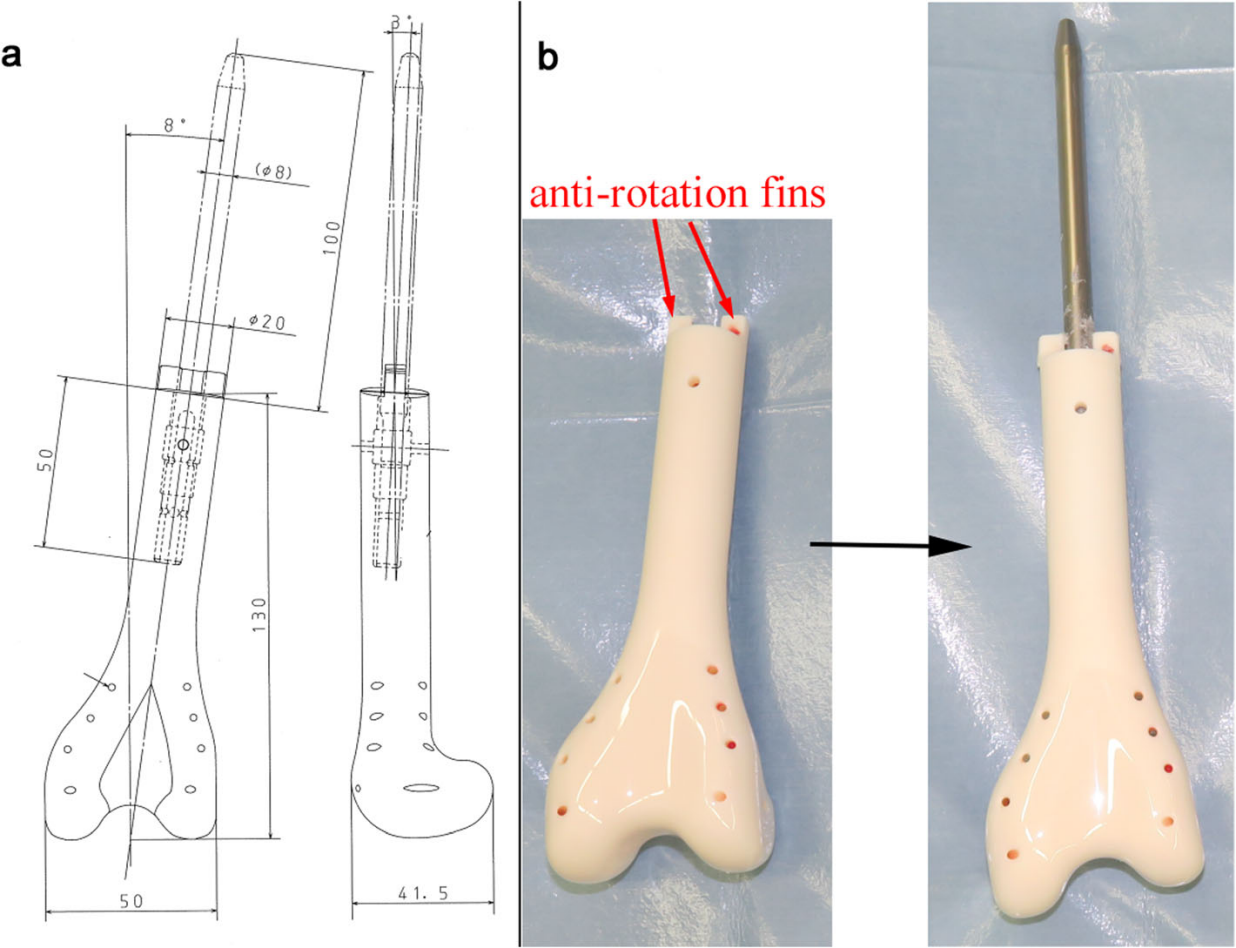
**a** The pre-production design and **b** photographs of the custom-made spacer

**Fig. 5:**
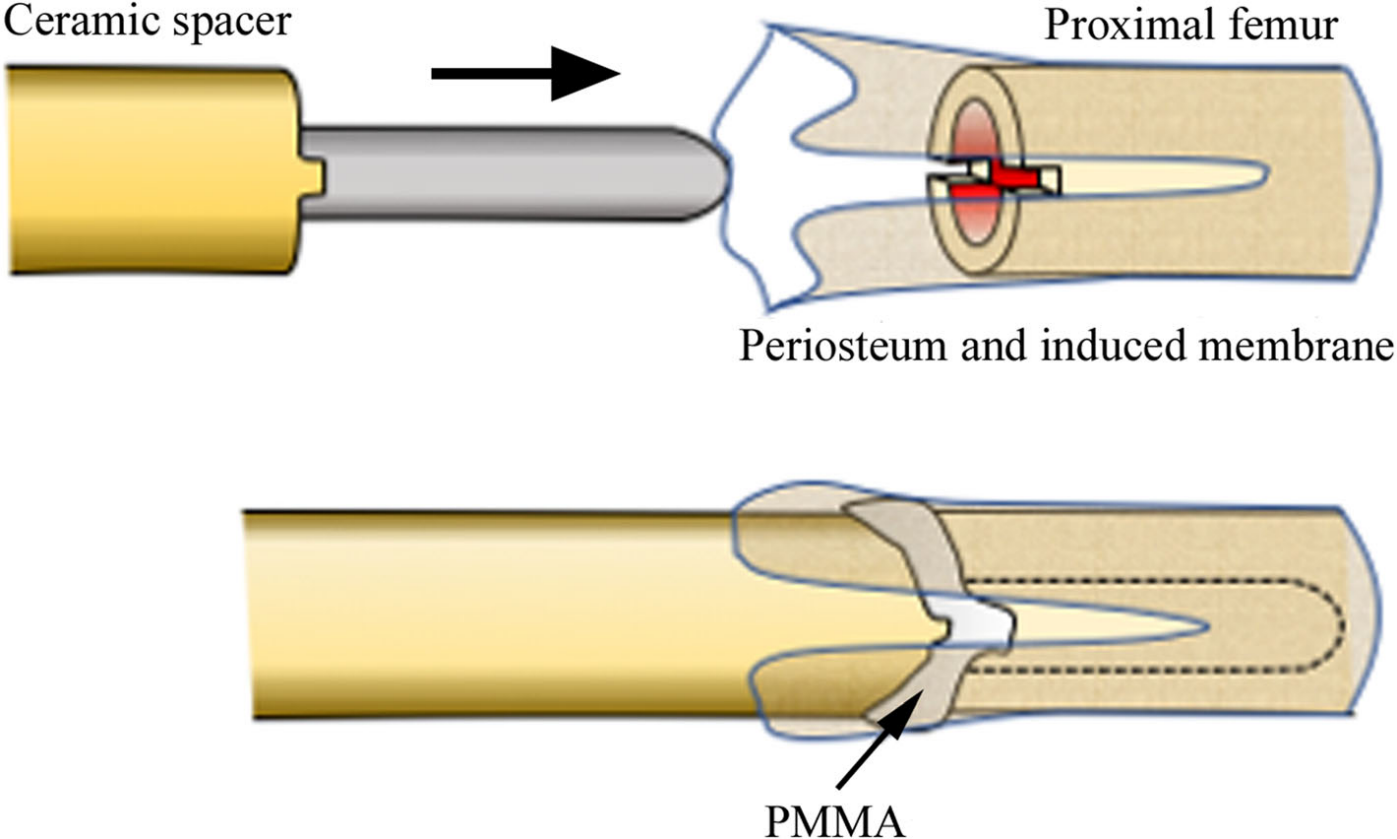
The scheme for “partial cementation and preservation of the periosteum”

**Fig. 6:**
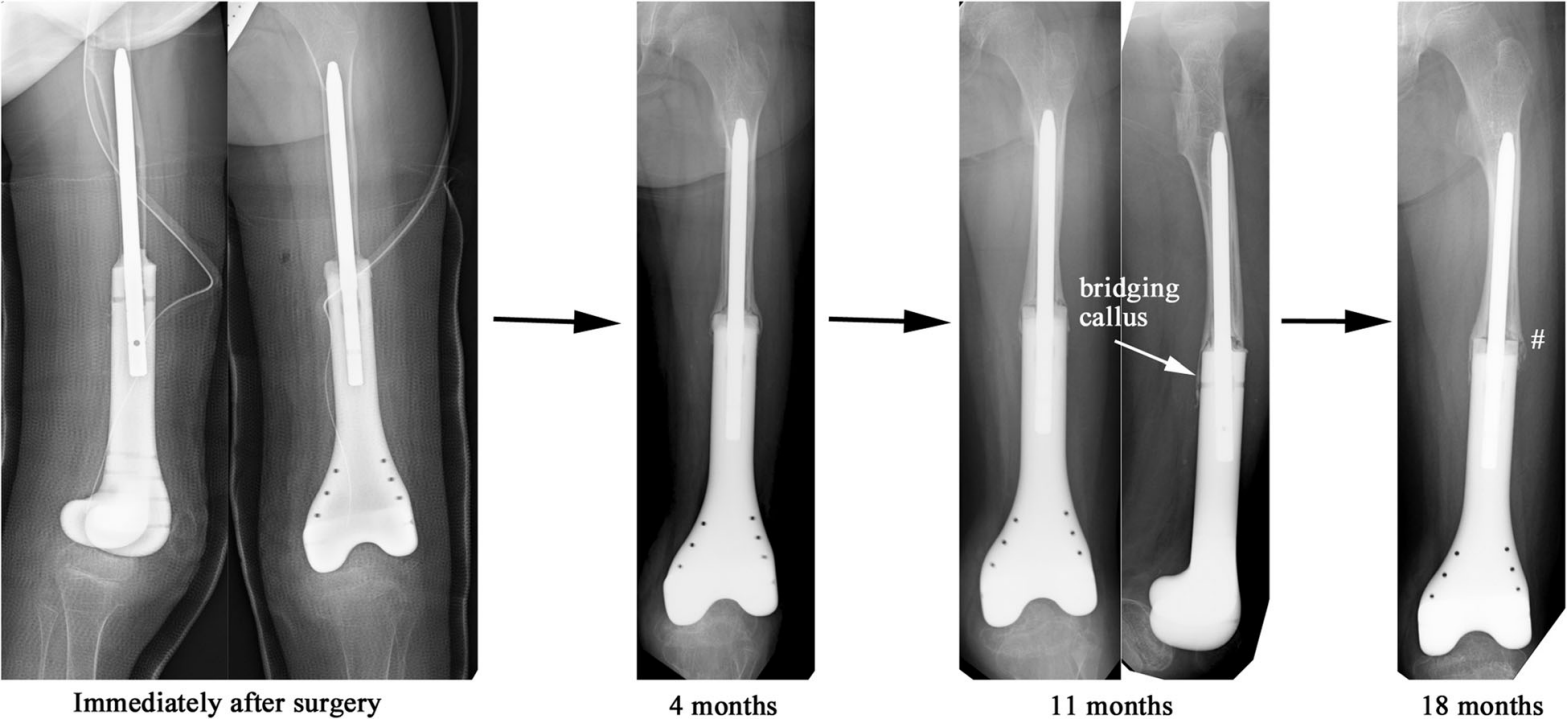
A series of radiographs after the second surgery. Compared to immediately after surgery, bone bridging was apparent at 4 months post-operatively, with femoral cortex thickening, a microfracture (#) and varus deformity observable at 18 months, indicating the loosening of the stem

At 7 years and 8 months old, secondary surgery was performed with the aim of enabling weight bearing on the affected leg. In order to stabilize the spacer against the tibia, a custom-made ceramic spacer (Kyocera Corp., Japan) with a smooth straight stem of 8 mm in diameter had been ordered 3 months prior to the surgery (Fig. 4a). Intra-operatively, after the removal of the initial spacer, a 1-cm length of the distal edge of the residual femur (Fig. 4b) was removed, but the periosteum and reactive membrane around the molded PMMA was preserved. The junction between the residual femur and the ceramic spacer was fixed with PMMA, and then covered with the preserved periosteum and membrane (Fig. 5). After surgery, the left leg was placed in a cast for 4 weeks, after which the patient re-started partial weight bearing using a hinged knee brace as with the first spacer. The bone atrophy in the left leg was gradually resolved. At 18 months after the second surgery, the cortexes of the femur and tibia had thickened sufficiently for expandable mega-prosthesis; however, loosening of the stem and varus deformity was observed (Fig. 6). A third surgical intervention was, therefore, planned.

**Fig. 7:**
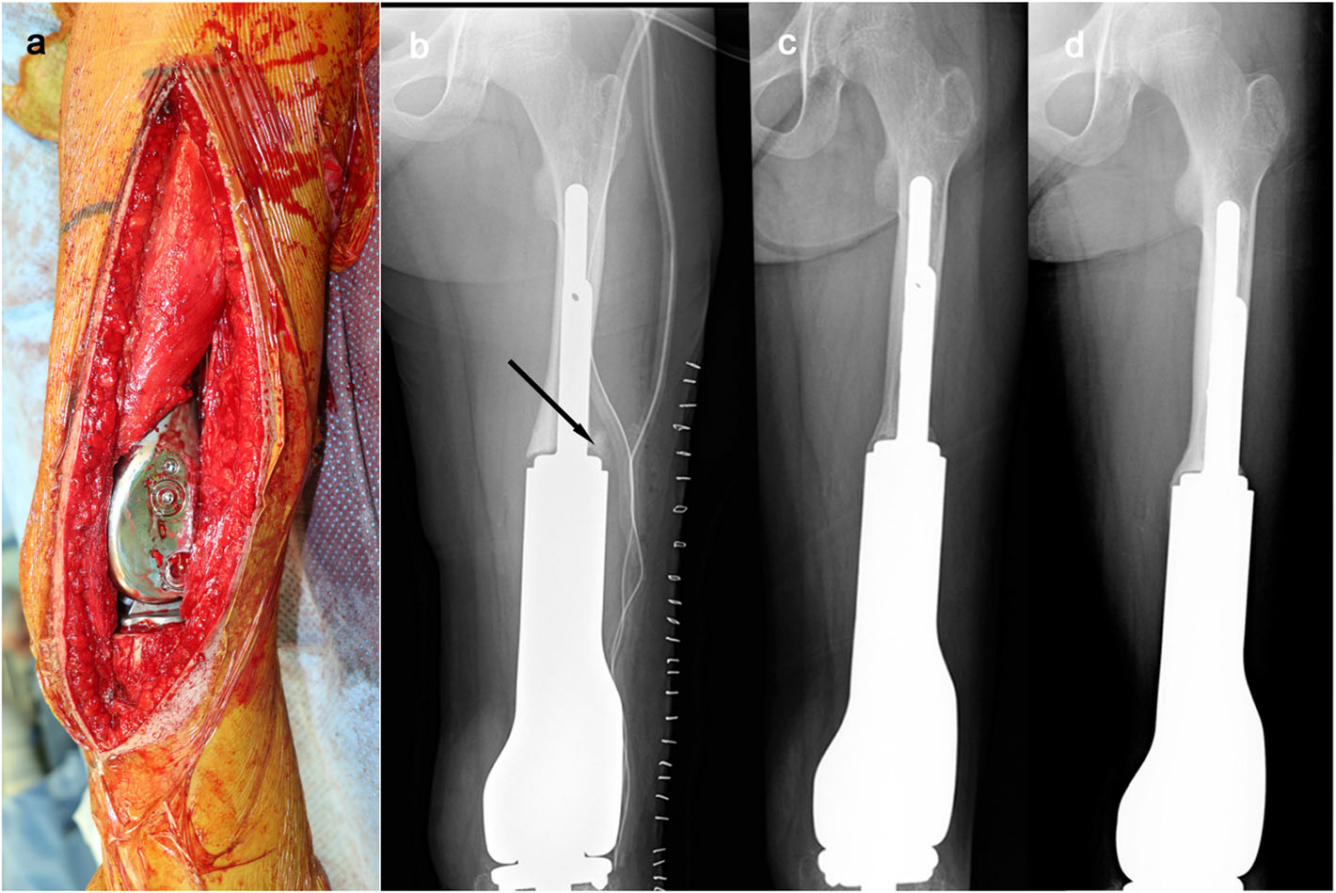
An intra-operative photograph of the third surgery (**a**) and radiographs immediately after surgery (**b**), 5 months post-operatively (**c**), and 10 months post-operatively (**d**). A black arrow points cancerous bone tips under the preserved periosteum and induced membrane

**Fig. 8:**
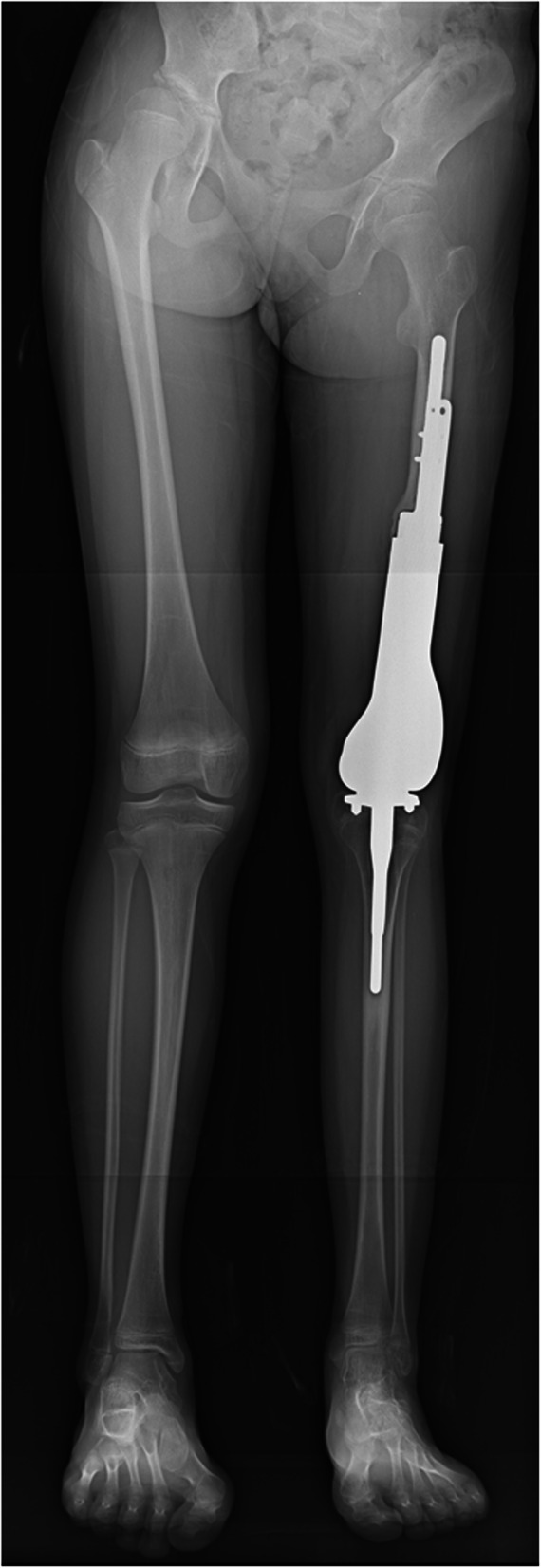
Lower limb full length x-ray at 11 years and 1 month. Although the ipsilateral tibia grew similarly to the contralateral side, the LLD was 5 cm. The expandable prosthesis was already extended by 1 cm

At 9 years and 7 months old, the second spacer was removed, and the knee joint was reconstructed using a custom-made growing femoral prosthesis (Stryker Corp., Germany) with a curved porous stem of 8.5 mm in diameter fixed to the residual femur with two screws (Fig. 7a). Intra-operatively, the PMMA around the junction was already loosened, and the stem was easily removed. The residual femur was carefully reamed, and the stem was inserted. Cancellous bone chips from the proximal tibial epiphysis were grafted around the bone-prosthesis junction and then wrapped with the preserved periosteum and pseudo-periosteum membrane. The proximal surface of the tibia was also replaced, but the growth plate was preserved. Immediately after surgery, range of motion (ROM) exercise was started. Six weeks after surgery, partial weight bearing was permitted. Ten months after the third surgery, the patient was able to walk unsupported, and a radiograph showed further thickening of the cortex of the residual femur (Fig. 7b–d). At 10 years and 7 months old, the growing femoral prosthesis was extended by 1.1 cm. At the latest follow-up, at 11 years and 1 month old, the patient was 143 cm in height with a limb length discrepancy (LLD) of 5 cm (Fig. 8) and was able to walk unsupported using a 2.5 cm shoe lift (Video 1). The Musculoskeletal Tumor Society (MSTS) score was 24 out of 30 points [8]. The muscle strength of quadriceps recovered to manual muscle test (MMT) level 3, and active straight leg raising became possible without extension lag. The ROM of the left knee was 0–90°. The patient was receiving a second extension of growing femoral prosthesis.

## Discussion and conclusions

Reconstruction after distal femoral resection for patients in early childhood is challenging. Rotationplasty is a viable reconstruction option, offering generally acceptable long-term functional outcomes [9], but this surgical procedure is not always optimal as it is not always accepted emotionally by patients and their guardians. Alternatively, an osteo-articular allograft can be used for reconstruction after distal femoral removal, but this procedure is susceptible to complications. In a retrospective study of allograft reconstructions for sarcoma patients younger than 10 years by Aponte-Tinao et al., four of the seven patients undergoing osteo-articular allograft reconstruction with long-term follow-up experienced major complications: two fractures, one infection, and one non-union [2]. In our case, mega-prosthetic reconstruction was not feasible due to the intramedullary diameter of the affected femur being too narrow, and rotationplasty was refused. As allograft supply is rather limited in Japan, we had to seek an alternative option to act as a bridge until mega-prosthetic reconstruction became feasible.

The use of a temporary spacer after the removal of the distal femoral tumor was first reported in 1982 by Volz et al., but their concept differed from ours. They used a temporary spacer for a 31-year-old woman with a recurrent malignant bone tumor as a stabilizing filler for only 13 weeks until an appropriate customized implant was prepared [10]. In 2015, Chung et al. published a case series of temporary hemiarthroplasty utilizing synthetic mesh for children with osteosarcoma around the knee. The authors performed hemiarthroplasty with or without an allograft in 12 children aged 8–12 years with distal femoral osteosarcoma, four of which eventually underwent non-expandable tumor prosthetic reconstruction 23–47 months after hemiarthroplasty. Synthetic mesh was used to reconstruct the soft tissue and stabilize the joint. They reported good clinical outcomes, with MSTS scores of 24–28 points and the most recent LLD of 1.0–4.6 cm at 29–76 months after the first surgery [5].

In our case, our first hand-made spacer was dislocated at 7 months post-operatively. Due to the misfitting of the first spacer to the proximal tibia, we chose a commercially available custom-made spacer (Fig. 4) for the second surgery. It was characterized by its fitting the anatomical shape of the distal femur, its smooth surface suitable for articulation, and an anti-rotation fin at the proximal end of its body. Due to the absence of anti-rotation screw(s) and looseness between the anti-rotation fin and the residual bone, we decided to partially fix the spacer using PMMA to prevent rotation at the bone-spacer junction. In addition, we sought to achieve the formation of an osseous bridge over the bone-spacer junction by covering the junction with the periosteum and induced membrane. Instability of the knee joint can be an issue without ligamentous reconstruction, but we did not use synthetic mesh as a fibrous pseudo-capsule had already formed at the time of the second hemiarthroplasty. After the second hemiarthroplasty, the patient could begin ambulation with weight bearing on the affected leg through use of hinged knee brace but without any other support, and this functioned well for 18 months, allowing periosteal bone formation over the junction and the successful mega-prosthetic reconstruction with insertion of a durable stem.

The use of PMMA may have played an essential part to achieve durable bone-prosthesis junction. In 2003, Masquelet reported 35 cases of long bone defect successfully treated with 2-step reconstruction: cement spacer insertion followed by autologous cancellous bone grafting. The concept is that spacer is responsible for the formation of synovium-like membrane, which can prevent grafted bone from resorption and favor its revascularization and corticalization [11]. Since then, “Masquelet technique” has become a standard treatment for long bone defect. In our case, we also preserved induced membrane around PMMA and grafted cancellous bone tips under them at the third surgery. After surgery, the cortex of the residual bone became satisfactorily thickened and periosteal/peri-membranous bone formation overlapped the bone-prosthesis junction. It is difficult to prove that such successful bone formation is attributed to cementation and following membrane formation, but we believe that temporal spacer accompanied by partial cementation is better than immediate prosthetic reconstruction in terms of bone formation around the bone-prosthesis junction.

The functional results of our case were slightly worse than those reported by Chung et al., but such inferior outcomes can mostly be attributed to the younger age of our case, along with the instability of the first spacer. The surgical procedure and post-operative management are obviously more difficult in younger patients due to their smaller bone size and expected longer LLD. Our case, however, indicates that hemiarthroplasty can be applied to younger children with malignant distal femoral tumor by utilizing the aforementioned surgical technique.

In conclusion, this case report provides an example of limb-salvage surgery after distal femoral resection in a small child. Along with allograft reconstruction, the use of a temporary spacer utilizing partial cementation and preservation of the periosteum and induced membrane after PMMA removal appears to afford a viable limb-salvage option after distal femoral resection for younger children refusing femoral rotationplasty.

## Supplementary Information

**Additional file 1: Video 1.** The patient was walking using a 2.5-cm shoe lift with a slight limb at 11 years and 1 month old, before the second extension of the expandable prosthesis.

## Data Availability

Not applicable

## Abbreviations

PMMA: Polymethylmethacrylate
ROM: Range of motion
LLD: Limb length discrepancy
MSTS: Musculoskeletal Tumor Society
MMT: Manual muscle test
